# Associations between the measures of physical function, risk of falls and the quality of life in haemodialysis patients: a cross-sectional study

**DOI:** 10.1186/s12882-019-1671-9

**Published:** 2020-01-06

**Authors:** Karsten Vanden Wyngaert, Amaryllis H. Van Craenenbroeck, Sunny Eloot, Patrick Calders, Bert Celie, Els Holvoet, Wim Van Biesen

**Affiliations:** 1 Department of Rehabilitation Sciences, Faculty of Medicine and Health Sciences, Ghent University, Corneel Heymanslaan 10, 9000 Ghent, Belgium; 2 Department of Nephrology, University Hospitals Leuven, Leuven, Belgium; 3 Laboratory of Experimental Medicine and Paediatrics, University of Antwerp, Antwerp, Belgium; 4 Department of Internal Medicine, Renal Division, Ghent University Hospital, Ghent, Belgium

**Keywords:** End-stage kidney disease, Physical function, Risk of falls, Haemodialysis, Quality of life

## Abstract

**Background:**

Impaired physical function due to muscle weakness and exercise intolerance reduces the ability to perform activities of daily living in patients with end-stage kidney disease, and by consequence, Health-Related Quality of Life (HRQoL). Furthermore, the risk of falls is an aggregate of physical function and, therefore, could be associated with HRQoL as well. The present study examined the associations between objective and subjective measures of physical function, risk of falls and HRQoL in haemodialysis patients.

**Methods:**

This cross-sectional multicentre study included patients on maintenance haemodialysis. Physical function (quadriceps force, handgrip force, Sit-to-Stand, and six-minute walking test), the risk of falls (Tinetti, FICSIT-4, and dialysis fall index) and HRQoL (PROMIS-29 and EQ-5D-3 L) were measured and analysed descriptively, by general linear models and logistic regression.

**Results:**

Of the 113 haemodialysis patients (mean age 67.5 ± 16.1, 57.5% male) enrolled, a majority had impaired quadriceps force (86.7%) and six-minute walking test (92%), and an increased risk of falls (73.5%). Whereas muscle strength and exercise capacity were associated with global HRQoL (R^2^ = 0.32) and the risk of falls, the risk of falls itself was related to psycho-social domains (R^2^ = 0.11) such as depression and social participation, rather than to the physical domains of HRQoL. Objective measures of physical function were not associated with subjective fatigue, nor with subjective appreciation of health status.

**Conclusions:**

More than muscle strength, lack of coordination and balance as witnessed by the risk of falls contribute to social isolation and HRQoL of haemodialysis patients. Mental fatigue was less common than expected, whereas, subjective and objective physical function were decreased.

## Background

Exercise intolerance, defined as decreased physical ability to perform activities requiring muscle strength and cardiovascular capacity, is common in patients on haemodialysis (HD) [1]. Furthermore, the combination of cardiovascular comorbidities and changes in muscle characteristics such as mitochondrial dysfunction lead to reduced oxidative contribution resulting in aerobic exercise intolerance [2, 3]. In addition, catabolic processes, inflammation and malnutrition further contribute to muscle wasting and physical inactivity in HD patients [4, 5].

Next to decreased muscle strength, uremic polyneuropathy, autonomic dysfunction, hypotensive episodes, and polypharmacy contribute to an increased risk of falls as well, resulting in a 4.4 times higher risk for hip fractures in HD patients compared to age-matched healthy control subjects [6, 7]. The risk of falls could be seen as an aggregate marker of physical function. Both reduced maximal oxygen uptake and an increased risk of falls are predictive for morbidity and mortality in patients on HD [8].

A reduced exercise capacity and muscle strength affects the ability to perform activities of daily living (ADL) [9], and hence potentially, the overall Health-Related Quality of Life (HRQoL) [8, 10]. However, the impact of the risk of falls on HRQoL on top of muscle weakness and exercise capacity has not yet been examined in HD patients. Nonetheless, this is an important question as it could result in different training and rehabilitation needs for this population [8].

Furthermore, fatigue is the common complication in patients on HD (73–91%) [11, 12] and was recently identified as the most important outcome by all stakeholders [13]. It is characterized as both a physical and psychological symptom, and has a multi-faced aetiology, ranging from plain physical exertion over physical performance and energy management to depression [14]. The association between fatigue and the components of HRQoL is potentially bilateral, as the lack of energy due to reduced physical capacity might induce depression and mood disorders, or it could be the other way around [15]. Again, better insight into the association between subjective fatigue and objective physical function is important, as it can steer interventional strategies.

We hypothesised that both psychosocial and physical domains of HRQoL are associated with objective measures of physical function in patients on HD.

The purpose of this cross-sectional study was to examine the association between the objective and subjective measures of physical function, the risk of falls and subcategories of HRQoL in prevalent HD patients.

## Methods

### Participants and study design

Patients on maintenance HD, who were included in a larger study (registration number on clinicaltrial.gov: NCT03910426), in two main dialysis centres were screened for eligibility between December 2016 and March 2018. These dialysis centres included five different dialysis units (two high-care and five low-care dialysis units) distributed throughout five different public hospitals. Exclusion criteria were the following: age < 18 years, pregnancy, active inflammation, malignancy, cognitive disorders, inadequate motor and verbal responses to verbal commands and questions, and recent (< 6 months) surgical musculoskeletal interventions. Patients with physical inabilities (e.g. wheelchair bound or amputations) were given the worst possible score for those examinations they failed to complete.

The study complies with the Declaration of Helsinki, was approved by the local ethics committees (project number Ghent EC B670201525559 and Antwerp EC B300201422642), and written informed consent was obtained.

### Anthropometric measures and characteristics

Baseline clinical data and anthropometric measures were obtained, and pre- and post-dialysis mean arterial pressures (MAP) were calculated as diastolic blood pressure + 1/3(systolic blood pressure - diastolic blood pressure). Blood pressures were measured with a single measurement at the upper-arm opposite to the vascular access.

### Physical examinations

The sequence of the different tests was randomized using opaque envelopes. Muscle strength examinations took place before dialysis sessions, while the measurements of functional exercise capacity and the risk of falls were examined either before dialysis or in patients’ home setting on non-dialysis days. A minimum 3-min pause between tests was respected and utmost care was taken that the different assessments of functional exercise capacity were not directly consecutive.

### Muscle strength

A handheld dynamometer (Microfet; Biometrics, Almere, the Netherlands) was used to evaluate quadriceps isometric peak torque in a sitting position with knees and hips 90° flexed [16]. Manual resistance with fixation of the dynamometer to the anterior tibia of the dominant leg just proximal to the malleoli was applied by a researcher for 5 s [17]. Handgrip force of the hand opposite to the vascular access was measured using a JAMAR Hydraulic Hand Dynamometer according to the American Society of Hand Therapists guidelines [18]. Patients were seated with their elbow 90° flexed next to their body, wrist in neutral position and were asked to perform a maximal isometric contraction for 5 s [19].

The best out of 3 attempts was expressed as absolute value and as the percentage of the predicted value based on age and gender [16, 20].

### Physical functioning

The six-minute walking test (6MWT) is a functional examination of exercise capacity and was performed following the American Thoracic Society guidelines [21]. Patients were instructed to walk as fast as possible for 6 min, walking aids were allowed and recorded. Results were expressed as absolute value and as percentage to the predicted value [22]. A 350 m cut-off point was used, as this indicates a worse prognosis and higher mortality in populations comparable to patients on HD [23, 24].

### The risk of falls

For assessment of the risk of falls, a combination of physical testing, scoring lists and demographic data was used in a slightly adapted version of the Dialysis Fall Risk Index (DFRI, Table 1) [25]. With regard to the original DFRI, the following adaptations were made: (1) a 2.9 mg/dl instead of 1.0 mg/dl cut-off point for C-reactive protein; (2) mini-nutritional assessment indication scores were used as an alternative for the Geriatric Nutritional Risk Index [26]; (3) 6MWT with an additional 300 m cut-off point for an increased risk of falls replaced the ‘4 meter time to walk’ test; and (4) the ‘inquiry about fall’ section was replaced by the Tinetti test [27].

**Table 1:** Dialysis risk of falls index

Topic	Check			Score
Age	≥80 years old			
	⎕ Yes			1.5
	⎕ No			0
Serum C-reactive Protein	≥3.0 mg/dL			
	⎕ Yes			2.0
	⎕ No			0
Risk for malnutrition	< 24 on 30			
	⎕ Yes			0.5
	⎕ No			0
Physical examinations	Standing balance			
	- Side-by-side stand			
	⎕ 10 s	⎕ 1		
	⎕ < 10 s	⎕ 0		
	- Semi-tandem stand			
	⎕ 10 s	⎕ 1		
	⎕ < 10 s	⎕ 0		
	- Full-tandem stand			
	⎕ 10 s	⎕ 2	⎕ < 8 points	2.5
	⎕ 3–9.9 s	⎕ 1		
	⎕ < 3 s	⎕ 0	⎕ 9–10 points	2.0
	6MWT			
	⎕ > 350 m	⎕ 4	⎕ 11–12 points	0
	⎕ 300–350 m	⎕ 2		
	⎕ < 300 m	⎕ 0		
	STS			
	⎕ < 11.19 s	⎕ 4		
	⎕ 11.20–13.69 s	⎕ 3		
	⎕ 13.70–16.69 s	⎕ 2		
	⎕ 16.70–49.99 s	⎕ 1		
	⎕ > 50 s or impossible	⎕ 0		
Handgrip force	Male < 26 kg, female < 18 kg			
	⎕ Yes			1.5
	⎕ No			0
Intra-dialytic hypotension	MAP decrease > 9.99 mmHg			
	⎕ Yes			1.5
	⎕ No			0
Risk of falls assessment	Tinetti < 11 on 12 points			
	⎕ Yes			2.5
	⎕ No			0
Total score				/12

*Abbreviations: 6MWT* six-minute walking test, *MAP* mean arterial pressure, *STS* sit-to-stand test

The Tinetti test is considered the gold standard for examining fall-related gait dysfunctions based on 7 items: initiation of gait, step length and height, step symmetry, step continuity, distinguished path, trunk and walking stance. Patients scoring < 11 on 12 on the Tinetti test are considered at a high risk of falls [28].

The Frailty and Injuries Cooperative Studies of Intervention Technique-4 (FICSIT) was used to examine static balance (on time) based on seven positional challenges; i.e. eyes open and closed with feet closely together, semi-tandem and full tandem stand, and standing on the dominant leg with eyes open [29]. No consistent cut-off values were found in the literature.

The five repetition Sit-to-Stand (STS) was used to assess the risk of falls as well as functional lower limb muscle strength [30]. Patients were instructed to get from a sitting to a standing position for 5 times as rapidly as possible with their arms folded across the chest [31]. A cut-off value of ≥15 s is associated with an increased risk of falls [32].

### Health-related quality of life

Using a Dutch version of the EQ-5D-3 L from the Euro QOL Group and the PROMIS-29 v2.0, patients were surveyed by a study nurse during the dialysis session closest to the physical examinations. The EQ-5D comprises five dimensions: mobility, self-care, usual activities, pain discomfort and anxiety-depression which are scored on a 3-point Likert scale [33]. The PROMIS questionnaire assesses following seven domains using four questions for each domain, which are scored from 1 to 5: depression, anxiety, physical function, pain interference, fatigue, sleep disturbance and ability to participate in social roles and activities. The PROMIS has been validated in patients with chronic diseases, albeit, to our knowledge, not in patients with end-stage kidney disease [34, 35]. However, as it is considered a generic scale of HRQoL, it should deliver reliable results as well.

### Statistical analysis

IBM Statistical Package for the Social Sciences version 24 was used for all statistical analyses. Descriptive analysis reports mean ± standard deviation (SD), median and interquartile range [25th; 75th percentage] or number and percentage when appropriate. The data of the PROMIS questionnaire are reported as T-scores based on a representative sample of the US population. Reference values of EQ-5D are based on a Belgian population of similar age and gender distribution [36]. The lower limit of normal (LLN) for the quadriceps and handgrip force, and 6MWT was set on 80% of the predicted value. Patients unable to perform the 6MWT and STS were scored as ‘0 m’ and ‘> 50 seconds’ respectively. Patients were distributed into three groups of global physical performance on regard of impairments in neither, only one or both the 6MWT and DFRI (i.e. good, impaired or severely impaired physical performance groups respectively). Data between groups were compared with univariate analysis of variance and the post hoc Scheffe’s test. General linear models and logistic regressions were applied to evaluate the association between the parameters of interest.

## Results

A total of 122 patients were enrolled in this study. Nine patients with missing data were excluded, giving a response rate of 93%, albeit six patients for missing data on QoL questionnaires and three patients on measures of physical function. There were no major differences between the excluded and included subjects.

**Fig. 1:**
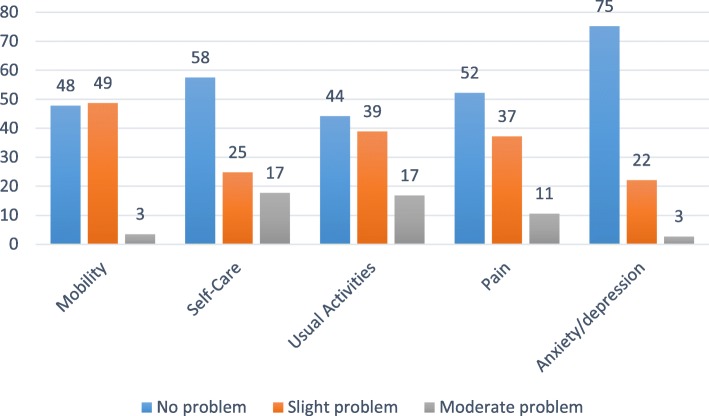
Problems reported on each domain of the quality of life. Data are reported as a percentage of the population

Table 2 presents data on patients’ characteristics and the parameters of interest. In this cohort (57.5% male, age 68 ± 16 years) cardiovascular disease was the most common comorbidity (74.3%) followed by diabetes (46.0%) and musculoskeletal complications (e.g. amputations and gout, 44.2%). In general, lower physical function and health utility than a presumed healthy, age-matched population were noted (see Additional file 1). Subjective difficulties with mobility and usual activities were reported by 52.2 and 55.8% of the cohort in the EQ-5D (Fig. 1). Decreased (> 1SD) appreciation of physical function and social participation according to the PROMIS was reported by 50.4 and 30.1% of the cohort. Furthermore, a minority reported significant complaints of pain (27.4%), depression (23.9%), fatigue (18.6%), anxiety (15.0%) and sleep disturbances (12.4%).

**Table 2:** Patients’ characteristics and parameters of interest

Variable	*n* = 113
Age (years)	68 ± 16
Male sex	65 (57.5)
BMI (kg/m^2^)	26.1 ± 5.4
Dialysis vintage (months)	22.5 [10.3; 49.8]
ΔMAP (mmHg)	0.17 ± 15.5
MAP pre-dialysis (mmHg)	89.7 ± 15.3
MAP post-dialysis (mmHg)	89.3 ± 17.8
Aetiology of CKD	
Glomerulonephritis	19 (16.8)
Hematologic malignancies	5 (4.4)
Interstitial nephritis	13 (11.5)
Diabetic nephropathy	30 (26.5)
Hypertension, angiosclerosis or unknown	40 (35.4)
ADPKD	6 (5.4)
Comorbidities	
Diabetes	52 (46.0)
CVD	84 (74.3)
Neuropathy	32 (28.3)
Retinopathy	36 (31.9)
Respiratory complications	28 (24.8)
Musculoskeletal complications	50 (44.2)
Hepatopathy	20 (17.7)
Quadriceps force	
Absolute value (N)	180 ± 75
Relative value (% to predicted)	53.8 ± 17.8
Handgrip force	
Absolute value (kg)	28.8 ± 11.1
Relative value (% to predicted)	91.7 ± 30.7
DFRI (/12)	5.9 ± 3.0
Q1	0–3.5
Q2	4–6.5
Q3	7–8.5
Q4	9–12
Tinetti (/12)	11.0 [5.5; 12.0]
Sit-to-Stand (s)	23.0 [12.0; 50.0]
FICSIT (/28)	15.0 [8.0; 21.0]
6MWT	
Absolute value (m)	236 [67; 397]
Relative value (% to predicted)	44.1 [12.7; 60.3]
EQ-5D	
Utility score	0.78 [0.41; 0.90]
Reference values based on age/sex	0.89 ± 0.01
VAS score (max 100)	58.8 ± 18.9
Reference values based on age/sex	78.3 ± 23.5
PROMIS (T-scores)	
Depression	51.2 ± 9.4
Anxiety	49.0 ± 9.0
Physical function	39.7 ± 11.1
Pain interference	51.1 ± 9.8
Fatigue	50.6 ± 10.1
Sleep disturbance	48.1 ± 9.2
Participation in social roles and activities	48.2 ± 10.0

Data are reported as mean ± standard deviation, median [25%; 75%] or as number (percentage) as appropriate

Abbreviations: *ADPKD* autosomal dominant polycystic kidney disease, *BMI* body mass index, *CVD* cardiovascular disease, *DBP* diastolic blood pressure, *MAP* mean arterial pressure, *SBP* systolic blood pressure, *ΔMAP* difference pre- to post-dialytic mean arterial pressure

**Fig. 2:**
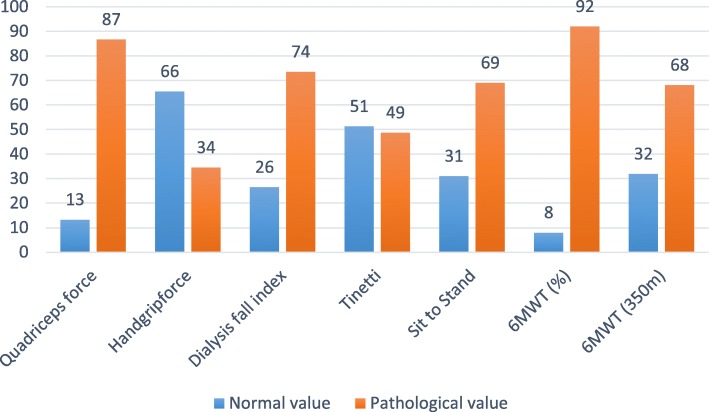
Physical impairments. Data are reported as a percentage of the population

Impairments in physical function were especially pronounced in lower limb muscle strength (86.7 and 69% had impaired values in absolute and functional quadriceps strength respectively) and in functional exercise capacity (92% scored below the LLN of the 6MWT, Fig. 2). A majority of studied patients scored below the 6MWTs’ clinically relevant cut-off point of 350 m (63.7%) and was rated with an increased risk of falls (73.5%). Noteworthy, the measures of the risk of falls were determined by objective measures of physical function (OR = 0.750), but were associated with functional exercise capacity more than muscle strength (see Additional file 2).

Although objective measures of physical function were barely associated with estimated personal health status (R^2^ = 0.05), the association between physical function and global HRQoL was modest and mainly driven by the 6MWT (R^2^ = 0.32, Table 3). Lower limb muscle strength and functional exercise capacity explained 51.6% of the variance in subjective physical function based on the EQ-5D. Despite an association between self-care and static balance, non-physical domains of the EQ-5D were not related to physical function (see Additional file 3). Regarding the PROMIS questionnaire, low associations were found between measures of the risk of falls and the appreciation of participation in social roles and activities on the one hand (R^2^ = 0.11), and depression on the other (R^2^ = 0.08). Additionally, a negligible relation was found between quadriceps strength and subjective fatigue. No measures of physical performance were associated with anxiety and sleep disturbance (Table 4).

**Table 3:** Association between the objective measures of physical function and EQ-5D

	EQ-VAS		EQ-5D score	
	F-value	*p*	F-value	*p*
Handgrip force (kg)	/	NS	6.52	0.012
Sit-to-Stand (s)	5.11	0.015	/	NS
6MWT (m)	/	NS	16.17	< 0.001
R square	0.053		0.318	

R square values are based on a general linear model; factors introduced to the model included absolute quadriceps and handgrip force, Sit-to-Stand test, 6MWT, Tinetti and the dialysis fall index. Abbreviations: *6MWT* six-minute walking test

**Table 4:** Association between the objective measures of physical function and PROMIS

Physical measures	Depression		Physical function		Pain interference		Fatigue		participation in social roles and activities	
	F-value	p	F-value	p	F-value	p	F-value	p	F-value	*p*
Quadriceps force (*N*)	/	NS	10.96	< 0.001	/	NS	4.83	0.03	/	NS
DFRI (/12)	6.54	0.01	/	NS	/	NS	/	NS	7.98	0.01
6MWT (m)	/	NS	42.21	< 0.001	4.56	0.04	/	NS	/	NS
R Square	0.078		0.516		0.039		0.044		0.111	

R square values are based on a general linear model; factors introduced to the model included absolute quadriceps and handgrip force, Sit-to-Stand test, 6MWT, Tinetti and the dialysis fall index. Abbreviations: *6MWT* six-minute walking test, *DFRI* dialysis fall risk index

**Fig. 3:**
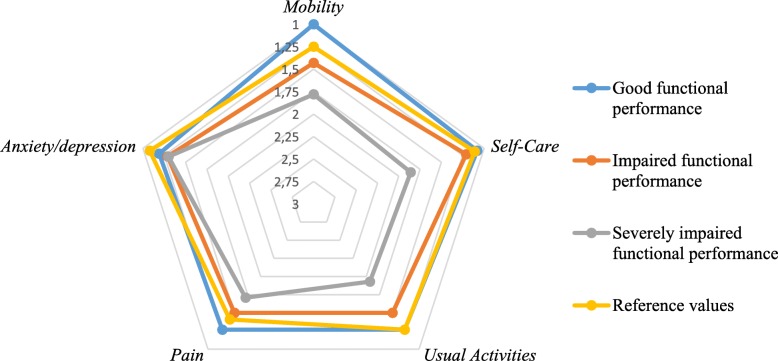
Radar chart of the dimensions of EQ-5D and functional performance. Increasing scores correspond with increasing difficulty in domains of HRQoL. Patients were allocated to the good, moderate or severely impaired functional performance group based on having no, only one of two or both unfavourable scores on 6MWT and dialysis fall index

After classifying patients based on global physical performance, patients with severely impaired functional performance scored worse on all domains of EQ-5D except for anxiety-depression (Fig. 3) and on subjective physical function assessed by PROMIS (see Additional file 1).

## Discussion

This study presents results of subjective and objective measures of physical function and their associations with HRQoL and health utility in patients on maintenance HD. To our knowledge, this is the first study to include risk of falls assessments to explore HRQoL and physical functioning in HD patients. We confirm a decreased HRQoL and health utility as well as decreased muscle strength and functional exercise capacity, and an increased risk of falls. Both muscle strength and functional exercise capacity were associated with physical domains of HRQoL and an increased risk of falls. Remarkably, the risk of falls on itself was identified as a determinant of difficulties on psycho-social well-being (i.e. depression and social isolation) and of objective health utility. Physical rehabilitation of HD patients aiming at improving HRQoL and health utility, should focus on improving coordination and functional capacity rather than purely on muscle strength and exercise capacity.

Adequate physical function is indispensable to be physically active. It requires both sufficient muscle strength and coordination to achieve adequate functional exercise capacity. Hence, impairments in one of those may induce difficulties in mobility and ADL. In addition, the capability to be self-supportive and to participate in society is determined by physical function as well [37]. Therefore, loss of physical function can have a substantial negative impact on psychological well-being, and as a consequence, on objective health utility and HRQoL. As improvements in functional exercise capacity by aerobic exercise training do not induce changes in physical or mental scores of HRQoL in patients on HD [38], a more comprehensive and multidisciplinary approach might be necessary to translate improvements in physical functioning in a positive change of HRQoL.

In uraemia, protein energy wasting and associated muscle weakness are common and relate to physical domains of HRQoL [39, 40]. Moreover, uraemia influences both cardiovascular and neurological systems, resulting in decreased exercise capacity and coordination [41]. These systems interact closely during functional activities, and it can thus be hypothesized that uraemia impacts functional exercise capacity not only by a decrease in muscle strength but also by decreased coordination.

Loss in proximal muscle strength as well as neuropathy and musculoskeletal comorbidities can lead to acute and chronic loss of postural stability and subsequently a high rate of falls [42]. Additionally, hypotensive episodes, anaemia, polypharmacy and impaired aerobic exercise capacity do not only cause early physical exertion during ADL, but also enhance the risk of falls [6, 25]. We found that muscle strength and especially functional exercise capacity explained variations in the risk of falls in HD patients. Consequently, an observed relation between the risk of falls and the physical domains of HRQoL could be expected. However, remarkably, the risk of falls related more to psycho-social than physical domains of HRQoL in the present study. As such, falls and an increased risk of falls can deter subjects to continue their outdoor social activities, resulting in changes in means and location of social contact to less stimulating activities (e.g. a phone call rather than a rendezvous point), promoting the risk of impairments in mental health and depression [43]. This hypothesis dovetails nicely with findings from a large Italian study on 227 HD patients that reported improvements in subjective quality of social interaction after an easy accessible physical activity program [44]. The provision of both physical and occupational therapy in HD patients should thus be considered as it could induce higher levels of physical activity, social participation and well-being. Especially prevention is of importance in HD patients as dialysis treatment hampers social integration and physical activity due to its time consuming impact [37, 45], and the high prevalence of reduced physical function, physical activity and HRQoL in this population [46, 47].

We found different associations between HRQoL and measures of physical function. Whereas objective HRQoL (EQ-5D score) was determined by absolute muscle strength, the appreciation of health status (EQ-VAS) showed only an association with functional muscle strength. The finding that patients accord more importance to functional than absolute measures of muscle function aligns with previous results that subjective health utility is associated with the accumulation of symptoms rather than the degree of one single symptom [48]. This discrepancy between objective measures of physical well-being and subjective health status has been addressed in other studies as ‘the well-being paradox’. [49]

Regarding fatigue, the PROMIS fatigue summary score is mainly a mental health factor (r = − 0.82) than a physical one (r = − 0.05) [50]. Mental fatigue as well as physical fatigue are identified as important complaints in HD patients worldwide. In our study, mental fatigue was scored not different from the general population, whereas impaired subjective physical function was much more expressed (50%). This suggests that complaints of fatigue in patients on HD are mainly due to subjective physical dysfunction [11, 12]. Subjective fatigue was associated with lower limb muscle strength.

Our study has some limitations. First, although we measured the risk of falls by multiple assessment tools, we have no history of actual falls. Instead, this study included the DFRI, a risk of falls assessment tool tailored for patients on HD. As this index was only published when our study was already ongoing, some alterations had to be implemented. Although these small adaptations could affect the general reliability compared to the original form, we used an equal item reliability to preserve the general reliability as well as possible. Second, the sample size of this study is small and does not exceed the rule of thumb of 20 subjects per variable, which is recommended by Green et al. However, the general linear model presented in this study has a subjects per variable-ratio of 19 and, thus, adequate estimations of regression coefficients, standard errors and confidence intervals can be performed [51].

A strength of this research was that we included HD patients willing to participate, without exclusion of the weakest, and this from multiple dialysis units. Hence, our research provides results that are applicable to the majority of populations on dialysis.

## Conclusions

We conclude that in this cohort of patients on maintenance haemodialysis the objective and subjective physical function, and Health-Related Quality of Life and health utility are decreased and that the risk of falls is increased. Particularly the risk of falls and functional exercise capacity explained large parts of decreased psycho-social and physical domains of the quality of life respectively. In contrast to available literature, subjective fatigue was less common than expected. Based on these findings, we advise health-care providers to include balance training and risk of falls prevention strategies in the standard care of patients on HD and as it could improve other domains of the quality of life compared to conventional exercise training.

## Supplementary information

**Additional file 1 Table S1:** Between-groups analysis of functional performance on the dimensions of quality of life.

**Additional file 2 Table S2 and S3:** Associations between the objective measures of physical function and the risk of falls.

**Additional file 3 Table S4:** Between-groups analysis of the subcategories of EQ-5D on physical function.

## Data Availability

As the full dataset is still being used for other analyses, it has not been anonymized yet. The datasets (without any identifying information) used and/or analysed during the current study are available from the corresponding author on reasonable request.

## Abbreviations

6MWT: Six-minute walking test
ADL: Activities of daily living
DFRI: Dialysis fall risk index
FICSIT: Frailty and injuries cooperative studies of intervention technique
HD: Haemodialysis
HRQoL: Health-related quality of life (HRQoL)
LLN: Lower limit of normal
MAP: Mean arterial pressure
STS: Sit-to-Stand
