# Supplementary material for: Associations between the measures of physical function, risk of falls and the quality of life in haemodialysis patients: a cross-sectional study
Source: BMC Nephrol. 2020 Jan 6;21:7. doi: 10.1186/s12882-019-1671-9 (PMC6945514; doi:10.1186/s12882-019-1671-9)
Supplement: Supplementary file 1 — Additional file 1 Table S1: Between-groups analysis of functional performance on the dimensions of quality of life. [file 12882_2019_1671_MOESM1_ESM.docx]

**Additional file 1.**

**Table S1: Between-groups analysis of functional performance on the dimensions of quality of life.**

| **Quality of life dimensions** | **Good functional performance (n=26)** | **Impaired functional performance (n=14)** | **Severely impaired functional performance (n=73)** | **P-value** | **Total (n=113)** | **Reference values** |
| --- | --- | --- | --- | --- | --- | --- |
| **Mobility** | 1.00 ± 0.00 | 1.43 ± 0.51 | 1.78 ± 0.53 | <0.001^a,b^ | 1.56 ± 0.57^c^ | 1.25 ± 0.12 |
| **Self-care** | 1.08 ± 0.27 | 1.21 ± 0.58 | 1.86 ± 0.80 | <0.001^d,e^ | 1.60 ± 0.77^c^ | 1.11 ± 0.08 |
| **Usual activities** | 1.27 ± 0.45 | 1.50 ± 0.65 | 1.93 ± 0.75 | <0.001^a^ | 1.73 ± 0.73^c^ | 1.27 ± 0.15 |
| **Pain/discomfort** | 1.27 ± 0.53 | 1.50 ± 0.52 | 1.71 ± 0.72 | 0.013^d^ | 1.58 ± 0.68^c^ | 1.41 ± 0.09 |
| **Anxiety/depression** | 1.19 ± 0.40 | 1.29 ± 0.47 | 1.30 ± 0.54 | 0.716 | 1.27 ± 0.50^c^ | 1.08 ± 0.01 |
| **PROMIS - depression** | 46.8 ± 8.72 | 52.1 ± 9.52 | 52.1 ± 9.32 | 0.058 | 51.2 ± 9.4 | N/A |
| **PROMIS - anxiety** | 46.8 ± 9.02 | 53.3 ± 10.7 | 50.2 ±8.37 | 0.077 | 49.0 ± 9.0 | N/A |
| **PROMIS - physical function** | 51.7 ± 6.63 | 44.3 ± 10.7 | 35.4 ± 9.05 | <0.001^e^ | 39.7 ± 11.1 | N/A |
| **PROMIS - pain interference** | 48.3 ± 7.97 | 53.7 ± 9.45 | 53.3 ± 10.2 | 0.056 | 51.1± 9.8 | N/A |
| **PROMIS - fatigue** | 48.0 ± 8.87 | 51.1 ± 8.59 | 51.2 ± 10.6 | 0.325 | 50.6 ± 10.1 | N/A |
| **PROMIS - sleep disturbance** | 48.2 ± 9.67 | 51.0 ± 8.30 | 48.6 ± 9.22 | 0.385 | 48.1 ± 9.2 | N/A |
| **PROMIS - participation and activities** | 49.6 ± 8.52 | 47.6 ± 9.77 | 44.8 ± 10.4 | 0.123 | 48.2 ± 10.0 | N/A |
| *Note*: data are reported as continuous measures, mean ± standard deviation and for the PROMIS-data as T-scores; p-values from Kruskal-Wallis test were reported; patients were allocated to a good, moderate or severely impaired functional performance group based on having no, only one of two or both unfavourable scores on 6MWT and dialysis fall index.  ^a^ p<0.05 good functional performance vs. moderate or severely impaired physical performance  ^b^ p<0.05 all three physical performance categories vs. reference values  ^c^ p<0.05 total cohort vs. reference values  ^d^ p<0.05 good functional performance vs. severely impaired physical performance  ^e^ p<0.05 good or moderate impaired physical performance vs. severely impaired physical performance | | | | | | |
