# Supplementary material for: Associations between the measures of physical function, risk of falls and the quality of life in haemodialysis patients: a cross-sectional study
Source: BMC Nephrol. 2020 Jan 6;21:7. doi: 10.1186/s12882-019-1671-9 (PMC6945514; doi:10.1186/s12882-019-1671-9)
Supplement: Supplementary file 2 — Additional file 2 Table S2 and S3: Associations between the objective measures of physical function and the risk of falls. [file 12882_2019_1671_MOESM2_ESM.docx]

**Additional file 2**

**Table S2: Association between the objective measures of physical function and the risk of falls.**

|  | **Tinetti** | | **FICSIT** | | **STS** | |
| --- | --- | --- | --- | --- | --- | --- |
|  | **F-value** | ***p*** | **F-value** | ***p*** | **F-value** | ***p*** |
| **Quadriceps force (N)** | / | NS | / | NS | 5.08 | 0.026 |
| **Handgrip force (kg)** | 5.92 | 0.017 | / | NS | / | NS |
| **6MWT (m)** | 143.16 | <0.001 | 159.57 | <0.001 | 71.55 | <0.001 |
| **R square** | 0.610 | | 0.590 | | 0.575 | |
| *Note*: R square values are based on a general linear model; factors introduced to the model included absolute quadriceps and handgrip force, and 6MWT  Abbreviations: 6MWT, six-minute walking test; STS, sit-to-stand | | | | | | |

**Table S3: Binary logistics physical performance on the risk of falls.**

|  | **Tinetti** | | **STS** | |
| --- | --- | --- | --- | --- |
|  | **Adjusted OR** | ***p*** | **Adjusted OR** | ***p*** |
| **Quadriceps force (N)** | / | NS | / | NS |
| **Handgrip force (kg)** | / | NS | 0.906 | 0.038 |
| **6MWT (m)** | 0.986 | <0.001 | 0.985 | <0.001 |
| **R square** | 0.665 | | 0.750 | |
| Nagelkerke R square values are presented, and a backward model was used; factors introduced to the model included absolute quadriceps, handgrip force and 6MWT  Abbreviations: OR, odds ratio | | | | |
