# Supplementary material for: Associations between the measures of physical function, risk of falls and the quality of life in haemodialysis patients: a cross-sectional study
Source: BMC Nephrol. 2020 Jan 6;21:7. doi: 10.1186/s12882-019-1671-9 (PMC6945514; doi:10.1186/s12882-019-1671-9)
Supplement: Supplementary file 3 — Additional file 3 Table S4: Between-groups analysis of the subcategories of EQ-5D on physical function. [file 12882_2019_1671_MOESM3_ESM.docx]

**Additional file 3**

**Table S4: Between-groups analysis of the subcategories of EQ-5D on physical function.**

| **Degree of trouble** | **n (%)** | **Quadriceps force (%)** | **Handgrip force (%)** | **Dialysis fall risk index** | **Tinetti** | **FICSIT** | **Sit-to-Stand (s)** | **6MWT (%)** |
| --- | --- | --- | --- | --- | --- | --- | --- | --- |
|  |  | **Mean ± SD** | **Mean ± SD** | **Mean ±SD** | **Mean ± SD** | **Mean ± SD** | **Mean ± SD** | **Mean ± SD** |
| **Mobility** | | | | | | | | |
| No  Moderate  Severe | 54  55  4 | 56.6 ± 17.8  51.6 ± 17.2  35.1 ± 23.2 | 92.1 ± 26.3  92.4 ± 34.2  77.4 ± 38.0 | **3.87 ± 2.70**  **7.59 ± 1.95**  **9.62 ± 1.03 ^b,f^** | **10.6 ± 3.26**  **6.58 ± 4.22**  **0 ± 0 ^b,f^** | **19.1 ± 7.96**  **10.8 ± 6.81**  **0 ± 0 ^b,f^** | **18.8 ± 13.9**  **39.6 ± 14.6**  **50.0 ± 0 ^b,f^** | **57.0 ± 27.1**  **26.8 ± 22.9**  **0 ± 0 ^b,f^** |
| **Self-care** | | | | | | | | |
| No  Moderate  Severe | 65  28  20 | 55.3 ± 17.8  52.4 ± 17.2  50.7 ± 19.1 | 89.1 ± 26.3  98.2 ± 38.0  90.5 ± 32.3 | **4.57 ± 2.76**  **7.23 ± 2.69**  **8.27 ± 1.91 ^b,f^** | **10.2 ± 3.24**  **7.21 ± 4.00**  **3.30 ± 4.40 ^b,f^** | **17.8 ± 7.97**  **13.2 ± 6.88**  **5.15 ± 6.29 ^c,f^** | **21.2 ± 15.0**  **38.8 ± 15.5**  **46.7 ± 8.66 ^b,f^** | **54.1 ± 26.7**  **29.3 ± 22.2**  **10.7 ± 19.0 ^b,f^** |
| **Usual activity** | | | | | | | | |
| No  Slight  Moderate | 50  44  19 | 56.8 ± 16.8  50.5 ± 17.7  52.7 ± 20.2 | 89.2 ± 22.0  95.6 ± 36.4  89.2 ± 35.9 | **4.36 ± 2.68**  **6.45 ± 2.80**  **8.57 ± 1.98 ^c,f^** | **10.2 ± 3.72**  **7.95 ± 3.96**  **4.05 ± 4.61 ^c,f^** | **17.8 ± 8.05**  **14.3 ± 7.85**  **5.84 ± 6.69 ^d,f^** | **20.7 ± 14.8**  **33.3 ± 17.4**  **46.9 ± 8.44 ^c,f^** | **55.2 ± 28.6**  **34.9 ± 24.5**  **13.3 ± 19.5 ^c,f^** |
| **Pain/discomfort** | | | | | | | | |
| No  Moderate  Severe | 59  42  12 | 55.5 ± 18.1  52.6 ± 17.1  49.3 ± 19.2 | 91.9 ± 29.6  93.2 ± 28.9  85.1 ± 43.2 | 5.33 ± 3.28  6.28 ± 2.52  7.21 ± 3.04 | 8.66 ± 4.64  8.05 ± 4.33  7.17 ± 4.39 | 15.4 ± 9.57  12.9 ± 7.67  15.1 ± 8.06 | **26.5 ± 18.1**  **33.8 ± 16.7**  **34.3 ± 17.1 ^e,f^** | 45.0 ± 32.6  38.1 ± 25.6  24.4 ± 23.0 |
| **Anxiety/depression** | | | | | | | | |
| No  Moderate  Severe | 85  25  3 | **56.4 ± 17.7**  **45.8 ± 14.9**  **34.1 ± 24.6  ^a,f^** | 93.9 ± 31.2  85.4 ± 28.0  81.6 ± 39.6 | 5.66 ± 3.04  6.22 ± 2.96  9.33 ± 3.03 | 8.72 ± 4.15  7.16 ± 5.34  5.00 ± 4.58 | 14.9 ± 8.24  13.1 ± 10.4  10.7 ± 9.24 | **27.6 ± 17.4**  **37.2 ± 17.1**  **39.7 ± 17.9 ^a,f^** | 42.8 ± 30.0  33.9 ± 28.9  20.4 ± 17.7 |
| *Note*: p-values from ANOVA were reported unless otherwise indicated (*), if so, they were reported from the Kruskal-Wallis test.  ^a^ p<0.05 group difference between no trouble vs. moderate trouble  ^b^ p<0.05 group differences between no trouble vs. moderate to severe trouble  ^c^ p<0.05 group differences between all three groups  ^d^ p<0.05 group difference between severe trouble vs. no to moderate trouble  ^e^ no between group differences were found based on adjusted p-values  ^f^ p<0.05 dichotomous group difference between no trouble vs. moderate and severe trouble | | | | | | | | |
